# Hyperbaric Oxygen Therapy for Cognitive Impairment Resulting from Neurological Disorders: Mechanisms of Action and Application Advances

**DOI:** 10.7150/ijms.128159

**Published:** 2026-08-24

**Authors:** Tianjiao Wei, Wenhui Gu, Lisha Ye, Guohua Wang

**Affiliations:** 1 Department of Neurology, Xuanwu Hospital Capital Medical University, Beijing 100053, China.; 2 Department of Neurophysiology and Neuropharmacology, Institute of Special Environmental Medicine and Co-innovation Center of Neuroregeneration, Nantong University, Nantong, Jiangsu 226019, China.; 3 Beijing Institute of Brain Disorders, Beijing 100069, China.

**Keywords:** hyperbaric oxygen therapy, cognitive impairment, neurovascular unit, neuroplasticity, neuroinflammation, mitochondrial dysfunction

## Abstract

Cognitive impairment is a common and disabling consequence of various neurological disorders. Due to its high prevalence, high conversion rate, and significant burden, it has increasingly become a major challenge for aging societies worldwide. Existing treatment strategies have limited efficacy in halting disease progression, making effective intervention strategies urgently needed. As a non-invasive physical therapy, hyperbaric oxygen therapy (HBOT) exerts its effects by increasing arterial oxygen partial pressure and improving tissue oxygenation. Historically widely used for treating carbon monoxide poisoning and decompression sickness, HBOT has recently demonstrated potential in alleviating symptoms of neurological disorders. Mechanistically, HBOT modulates the hypoxic microenvironment, promotes angiogenesis, restores neurovascular integrity, enhances neurogenesis and synaptic plasticity, and reduces neuroinflammation and oxidative stress. This paper systematically reviews the mechanisms of action, application progress, and existing limitations of HBOT in neurological disorders causing cognitive impairment. As a promising therapeutic modality, HBOT holds the potential to overcome current treatment limitations and become a key component of comprehensive neurological rehabilitation strategies.

## Introduction

Cognitive function constitutes the core capacity for perceiving, processing, and storing information, thereby enabling decision-making and behavioral regulation. It encompasses multiple higher-order mental processes including attention, memory, and executive function 1^.^ Its normal maintenance relies upon efficient functional interactions between neural networks within the brain 2. With the acceleration of global population ageing, the incidence and disability rates of cognitive impairment caused by neurological diseases such as Alzheimer's disease, vascular cognitive impairment, and traumatic brain injury continue to rise, presenting a severe global public health challenge 3. Such impairments not only severely diminish patients' quality of life but also impose substantial economic and caregiving burdens on families and society 4. Although the etiologies of cognitive impairment are complex and diverse, recent research has progressively revealed an underlying common core pathophysiological pathway: "cerebral tissue hypoxia - neurovascular unit imbalance - neural network degeneration" 5-7. Hypoxia compromises blood-brain barrier integrity and induces neuroinflammation and oxidative stress, leading to neural network dysfunction 8. Consequently, enhancing cerebral tissue oxygenation and restoring neurovascular unit function represent crucial strategies for alleviating cognitive impairment. Current treatments predominantly target downstream pathological products, merely delaying progression rather than reversing it. Hyperbaric oxygen therapy, a non-invasive physical modality targeting the upstream common pathological pathway of hypoxia, has garnered significant attention in recent years due to its cost-effectiveness and non-invasive nature 9. Hyperbaric oxygen therapy involves patients breathing pure oxygen at pressures exceeding 1 absolute atmosphere within a chamber to achieve therapeutic effects 10. It significantly elevates arterial oxygen partial pressure and tissue oxygen diffusion capacity 11, thereby fundamentally ameliorating cerebral hypoxia. Furthermore, it holds promise for remodelling neural plasticity through multidimensional, synergistic regulation of angiogenesis, neuroregeneration, inflammatory responses, and mitochondrial function 12. Therefore, in-depth exploration of hyperbaric oxygen therapy 's mechanisms of action holds significant promise for ameliorating cognitive impairment. This review examines the pathological features and therapeutic challenges of cognitive impairment arising from neurological disorders, elucidates hyperbaric oxygen therapy 's physiological properties, mechanisms of action, therapeutic advances, and limitations, thereby providing novel perspectives and strategies for advancing hyperbaric oxygen therapy 's role in improving cognitive dysfunction.

## Pathological Characteristics and Current Treatment of Cognitive Impairment

Cognitive impairment, as a core manifestation of multiple neurological disorders, is characterized by progressive decline in cognitive functions. This includes diminished attention and memory, impaired judgement, reasoning and abstract thinking, alongside restricted executive functions 13. Underlying these functional deficits lie distinct neuropathological alterations specific to different diseases. As a highly oxygen-demanding organ, the brain exhibits particular sensitivity to hypoxia 14. When brain tissue is damaged, microcirculatory impairment and reduced cerebral perfusion lead to a sharp decrease in oxygen supply. This subsequently triggers metabolic suppression, synaptic dysfunction, and ultimately neuronal death, forming the common metabolic basis for multiple cognitive disorders 15.

Alzheimer's disease is the most prevalent neurodegenerative disorder, characterized by core pathological features including cortical atrophy, cholinergic neuron loss, β-amyloid deposition, and neurofibrillary tangles resulting from hyper-phosphorylated Tau protein 16. These pathological alterations collectively cause neuronal disconnection and functional loss, clinically manifesting as progressive memory impairment, cognitive decline, and behavioral abnormalities, ultimately progressing to global dementia 17. Research indicates that cerebral hypoperfusion in specific regions, potentially triggered by neurovascular unit dysfunction or dysregulation of the brain's immunomodulatory network, may represent a latent precipitating factor for pathology of Alzheimer's disease. Furthermore, vascular dysfunction exacerbates Tau pathology, thereby worsening cognitive deficits 18. Traditional medications (such as cholinesterase inhibitors donepezil, rivastigmine, and memantine, and glutamate receptor antagonist memantine) alongside recent anti-Aβ monoclonal antibodies (aducanumab, donanemab), Tau inhibitors 19 (lecanemab), and stem cell therapies 20 and neuromodulation techniques (such as deep brain stimulation, transcranial magnetic stimulation 21) among other novel strategies remain unable to halt disease progression. The development of effective curative interventions is urgently required.

Vascular cognitive impairment arises from cerebrovascular lesions and associated risk factors, presenting a broad spectrum of cognitive impairment ranging from subjective cognitive decline to vascular dementia 22. Its core pathological mechanism involves chronic cerebral hypoperfusion causing oxygen and nutrient supply deficits, leading to cerebral ischemia, microinfarcts, and white matter lesions 23. Unlike Alzheimer's disease, which primarily manifests as episodic memory impairment, vascular cognitive impairment more typically presents with deficits in attention, judgement, reasoning, and executive function. Specific treatments remain lacking; clinical management relies primarily on controlling vascular risk factors, pharmacological interventions, and lifestyle modifications. Early identification and intervention are recognized as a critical window for delaying or even reversing cognitive decline 24.

The pathological basis of post- traumatic brain injury cognitive impairment primarily encompasses: 1 primary diffuse axonal injury caused by external force, disrupting white matter fiber connectivity; 2 microcirculatory impairment resulting from secondary intracranial hemorrhage and cerebral oedema; 3 disruption of the blood-brain barrier and subsequent activation of persistent neuro-inflammation 25. These alterations obstruct neural network information transmission, clinically manifesting as inattentiveness, diminished working memory, and slowed information processing 26. Although some patients recover within weeks, a significant proportion experience persistent cognitive impairment 27. Current cognitive therapies for traumatic brain injury primarily rely on a combined strategy of cognitive rehabilitation training, pharmacological interventions (such as methylphenidate and amantadine), and lifestyle adjustments. However, the long-term efficacy of medications remains controversial, necessitating the development of effective and sustained treatment protocols.

Despite differences in the etiology and initial pathological processes of these diseases, their downstream pathological cascades exhibit substantial convergence at hypoxia-related mechanisms. Chronic cerebral hypoperfusion and impaired oxygen delivery disrupt metabolic homeostasis, leading to mitochondrial dysfunction and energy insufficiency. This metabolic impairment subsequently compro-mises synaptic transmission and neurovascular coupling, triggers neuroinflammation and oxidative stress, and ultimately results in large-scale neural network dysfunction and cognitive decline. Therefore, therapeutic strategies targeting hypoxia and its associated pathological cascades may exert broad effects by simultaneously improving cerebral blood flow, restoring metabolic balance, and promoting neuroplasticity, thereby addressing multiple pathological components underlying cognitive impairment.

## The Physiological Basis of Hyperbaric Oxygen Therapy, Time-Dose-Dependent Effects, and Risk Management

Hyperbaric oxygen therapy has a long history, dating back to the early practices of British physician Henshaw in 1662. It is now approved by the Undersea and Hyperbaric Medical Society for 14 indications, including decompression sickness, air embolism, and carbon monoxide poisoning 28,29. Increasing evidence indicates that hyperbaric oxygen therapy demonstrates encouraging benefits across multiple systemic diseases 30-33.

Oxygen is a critical rate-limiting factor for brain function. When the brain performs various cognitive tasks, the regional oxygen consumption rate increases rapidly and specifically—this dynamic change constitutes the material basis of cognitive function 34. An adequate oxygen supply ensures efficient cognitive performance; conversely, hypoxia selectively impairs higher cognitive functions 35. Brain oxygen metabolism exhibits a high degree of tissue specificity and functional dependence: gray matter, rich in neuronal cell bodies and dense synapses and responsible for high-frequency signal integration and computational tasks, has a significantly higher oxygen consumption rate than white matter 36. Specific brain regions, such as the prefrontal cortex (responsible for language and multiple higher-order cognitive functions), are exceptionally sensitive to energy supply and exhibit extremely high metabolic demands 37. Furthermore, abnormal cerebral oxygen metabolism often precedes structural brain atrophy and can serve as an early functional biomarker of cognitive decline 38. Therefore, monitoring cerebral oxygen metabolism is crucial for understanding brain mechanisms and the pathological processes of various neurological disorders, such as stroke and Alzheimer's disease.

Hyperbaric oxygen therapy can improve memory, attention, executive function, and information processing speed; its physiological basis is grounded in Henry's Law and Fick's Law 39. Henry's Law states that the concentration of a solute gas in a solution is directly proportional to its partial pressure. Inhaling pure oxygen at 3 absolute atmospheric pressures (ATA) can cause the mean arterial blood oxygen partial pressure to surge from approximately 100 mmHg at normal atmospheric pressure to about 2000 mmHg; while the concentration of physically dissolved oxygen in plasma rises sharply from approximately 3 mL/L to 60 mL/L. This substantial increase in both oxygen partial pressure and oxygen content not only surpasses the physiological limits of oxygen transport at atmospheric pressure but also satisfies the basal oxygen requirements of all body tissues even without relying on hemoglobin 40 .According to Fick's law, the rate of gas diffusion through biological membranes (such as the alveolar-capillary membrane, and the capillary-tissue cell membrane) is directly proportional to the partial pressure difference across the membrane and inversely proportional to the membrane thickness. The massive oxygen partial pressure gradient created by hyperbaric oxygen therapy dramatically increases the partial pressure difference between capillaries and tissues, thereby significantly enhancing the rate of oxygen diffusion 41. When the distance between cells and capillaries increases due to edema, inflammation, or injury, hyperbaric oxygen therapy can overcome diffusion barriers, effectively expand the oxygen diffusion radius, improve oxygenation in edematous areas and neural tissues, correct focal hypoperfusion, and meet the metabolic demands of neurons 42 .These unique physiological advantages provide a solid theoretical foundation for the application of hyperbaric oxygen therapy in the treatment of cognitive impairments associated with neurological disorders.

However, this “hyperphysiological” oxygen supply inevitably leads to electron leakage in the mitochondrial electron transport chain, generating large amounts of reactive oxygen species (ROS) 43. Under normal physiological conditions at atmospheric pressure, the endogenous antioxidant enzyme system is sufficient to scavenge baseline levels of ROS and maintain redox balance. However, in a hyperbaric oxygen environment, once the pressure or duration of a single exposure exceeds a certain threshold, the instantaneous rate of ROS production will exceed the scavenging capacity of the antioxidant system. At this point, excess ROS no longer acts as a signaling molecule to induce adaptive protection but instead directly attacks membrane lipids, proteins, and DNA, triggering oxidative stress damage 44. This “oxidative stress threshold” in neural tissue constitutes the biological basis for the central nervous system's susceptibility to toxic damage from hyperbaric oxygen therapy, precise regulation of ROS is central to optimizing hyperbaric oxygen therapy strategies. Consequently, hyperbaric oxygen therapy exerts a biphasic regulatory effect on the central nervous system, and its therapeutic efficacy exhibits a strict time-dose relationship. Specifically: Short-term exposure (single session, < 2 h): Primarily exhibits antioxidant and anti-edema effects. Intermediate-term exposure (days to weeks): Induces angiogenesis and neuroplasticity. Long-term exposure or excessive pressure (> 3 ATA): Produces toxic effects. When pressure exceeds 4 ATA or a single session lasts longer than 3 hours, hyperbaric oxygen therapy can be used as a tool to induce oxidative stress, primarily causing central nervous system toxicity (such as oxygen-induced seizures) as well as lung and retinal damage (peripheral oxygen toxicity). Adverse reactions such as middle ear barotrauma and transient visual changes may also occur during treatment 45,46.

An intermittent hyperbaric oxygen exposure strategy allows for the partial clearance of ROS during intervals while maintaining effective peak oxygen partial pressure, thereby enhancing safety without compromising therapeutic efficacy 47 .The mechanism by which intermittent hyperoxic fluctuations activate HIF-1α follows the “hyperoxia-hypoxia paradox”: ROS bursts during hyperoxic periods persist into the intermittent phase, where they inhibit prolyl hydroxylase activity through oxidation, thereby blocking the oxygen-dependent degradation of HIF-1α and “mimicking” hypoxic signals during the reoxygenation phase 48. HIF-1α typically downregulates HO-1 induction; however, when Nrf2 is overexpressed, the inhibitory effect of HIF-1α is reversed, thereby promoting HO-1-mediated actions. This initiates antioxidant protective mechanisms to counteract hypoxia and activates regenerative repair mechanisms in damaged brain regions 49.

Regarding the safety window for hyperbaric oxygen therapy in the treatment of central nervous system disorders, although existing clinical studies have not established a completely uniform numerical standard, most evidence supports the following: within a pressure range of 2.0-2.5 ATA, with a single exposure duration of 60-90 minutes, once daily, five times weekly, for 30-40 consecutive sessions, an optimal balance between efficacy and safety can be achieved 50-55. However, the optimal time-dose combination still requires further standardization.

The safety of hyperbaric oxygen therapy is built upon systematic management of potential risks. These risks are directly correlated with treatment parameters (pressure, single session duration, cumulative sessions). Therefore, rigorous risk management is paramount in clinical practice, requiring adherence to multi-tiered safety protocols 56: 1) Adherence to the dose window: Employ individualized pressure settings (typically ≤ 2.5 ATA) and intermittent oxygenation protocols to mitigate the risk of oxygen toxicity; 2) Strict patient screening: Exclude contraindications such as active pneumothorax or severe emphysema; 3) In-chamber monitoring: Professional supervision with real-time vital sign monitoring and emergency protocols (e.g., immediate oxygen cessation and decompression upon seizure warning signs); 4) Adhere to authoritative guidelines: All procedures comply with the safety protocols of the Undersea and Hyperbaric Medical Society (UHMS) 57. Through these comprehensive measures, therapeutic benefits can be maximized while minimizing risks, providing critical reference value for the safe implementation of this technology. Therefore, further exploration of the mechanisms underlying hyperbaric oxygen therapy holds promise for offering new hope to patients with cognitive impairment.

### Mechanisms of action of hyperbaric oxygen therapy

As an adaptive function of the brain, impaired neuroplasticity disrupts cognitive function. Hyperbaric oxygen therapy is not merely "oxygen supplementation"; it promotes neuroplasticity through multiple mechanisms, thereby inducing cognitive improvement. These mechanisms span multiple levels, primarily encompassing: promoting cerebral angiogenesis and improving cerebral blood flow; inducing neuroregeneration and synaptic remodeling; regulating neuroinflammation; alleviating oxidative stress; and enhancing mitochondrial function 58-60** (Table 1)**.

#### Hyperbaric oxygen therapy promotes cerebral vascular repair and improves cerebral blood flow

The integrity of the blood-brain barrier and normal cerebral blood flow are prerequisites for maintaining homeostasis in the central nervous system and neural tissues 61. Pathological stimuli such as hypoxia and inflammation can disrupt the blood-brain barrier. In such cases, cytokines such as vascular endothelial growth factor (VEGF) promote angiogenesis by activating the migration and proliferation of endothelial cells 62,63. Studies have confirmed that hyperbaric oxygen therapy can maintain the integrity of the blood-brain barrier, dilate blood vessels to increase cerebral blood flow, and reduce cerebral edema and necrosis 64. Simultaneously, it activates nitric oxide synthase to elevate NO levels, reduces prolyl hydroxylase activity to stabilize HIF-1α, and consequently increases VEGF and bFGF levels, thereby accelerating angiogenesis 65. New blood vessels can alleviate local hypoxia, promote neurogenesis and synapse formation, induce brain plasticity, and improve cognitive function 66.

#### Hyperbaric oxygen therapy promotes neuroregeneration and synaptic plasticity restoration

Following central nervous system injury, hyperbaric oxygen therapy leverages hyperoxia and hyperbaric pressure to modulate oxygen-sensitive and pressure-responsive genes, increasing stem cell mobilization within tissues and creating favorable conditions for neuroregeneration 67. As a key regulator of the regenerative process, mTOR is a target of the PKB/AKT pathway, participating in stem cell maintenance, proliferation and differentiation, growth, cell survival, and autophagy 68. Hyperbaric oxygen activates the autophagy process by inhibiting the activation of the mTOR pathway, regulating p62, Beclin-1 and LC3 expression, promoting autophagosome formation and lysosomal fusion, and clearing toxic protein aggregates 69. Concurrently, hyperbaric oxygen therapy restores the balance between the anti-apoptotic Bcl-2 and the pro-apoptotic Bcl-2-associated X protein (BAX) to reduce neuronal apoptosis and promote neuronal recovery 70.

Synaptic plasticity constitutes the cellular basis of learning and memory, Brain-derived neurotrophic factor (BDNF) and cAMP response element-binding protein (CREB) are key regulators of synaptic plasticity and memory formation in the hippocampus 71. BDNF levels correlate significantly with performance in verbal memory, working memory, processing speed, and verbal fluency 72. Hyperbaric oxygen significantly promotes long-term potentiation (LTP) in the hippocampus of healthy rats by activating the BDNF/p-CREB pathway, and also enhances the long-term maintenance of LTP 73. Hyperbaric oxygen -induced BDNF upregulation drives synaptic enhancement by activating TrkB receptors. TrkB receptor activation engages MAPK/ERK and PI3K/Akt cascades, leading to CREB phosphorylation and promoting transcription of plasticity genes—including BDNF itself—thereby facilitating synapse formation and enhancing transmission efficacy 74, thereby preserving more brain tissue to maintain and enhance neural network function and significantly improve learning and memory capabilities 75.

As the white matter structure connecting the left and right cerebral hemispheres, the integrity of the corpus callosum influences information processing speed and episodic memory 76. Prolonged hyperbaric oxygen therapy enhances cerebral blood flow through improved oxygenation, increases macrophage activity to clear myelin debris, thereby activating Schwann cells to promote neurotrophic factor release, ultimately accelerating axonal and myelin regeneration 77,78. Crucially, hyperbaric oxygen therapy not only facilitates the initial stages of regeneration but also accelerates the repair process over time 79.

#### Hyperbaric oxygen therapy alleviates neuroinflammation

A complex interplay exists between the immune system and the central nervous system. When the blood-brain barrier is compromised, inflammatory mediators and immune cells enter the central nervous system, exacerbating neuroinflammation. This neuroinflammation, in turn, further compromises the integrity of the blood-brain barrier, creating a vicious cycle 80. Microglia, the primary immune cells of the central nervous system, undergo excessive activation. This triggers the release of pro-inflammatory cytokines such as IL-1β, IL-6, and TNF-α, leading to pathological synapse reduction, heightened oxidative stress, and cascading reactions that disrupt neural network function and accelerate cognitive decline 81. Research indicates that hyperbaric oxygen therapy can repair the damaged blood-brain barrier, inhibit abnormal proliferation of astrocytes and microglia, and increase expression of anti-apoptotic proteins such as Bcl-2 82,83. At the molecular level, the hyperoxic environment generated by hyperbaric oxygen therapy helps stabilize IκBα, suppressing activation of the NF-κB signaling pathway and thereby inhibiting pro-inflammatory cytokine production at the transcriptional level 84.

#### Hyperbaric oxygen therapy alleviates oxidative stress and improves mitochondrial function

The majority of the brain's oxygen metabolism is derived from glucose via oxidative phosphorylation within mitochondria. Reactive oxygen species are by-products of this process and, at physiological levels, participate as signaling molecules in initiating various biological processes such as cell death, survival, and differentiation 85. Oxidative stress occurs when reactive oxygen species production exceeds the clearance capacity of antioxidant systems, disrupting the redox equilibrium between free radicals and antioxidants within the body. Highly reactive free radicals not only induce damage such as cell membrane lipid destruction, protein and DNA cleavage 86, but also lead to mitochondrial DNA mutations, lipid peroxidation, and functional impairment 87. Hyperbaric oxygen therapy enhances the body's total antioxidant capacity by boosting the activity of antioxidant enzymes such as superoxide dismutase 1/2 and glutathione peroxidase, thereby reducing reactive oxygen species levels 88.

Mitochondria serve as the primary hub for oxidative stress in neurons, making them the principal molecular target of hyperbaric oxygen therapy. Research indicates that the hyperoxic state during hyperbaric oxygen therapy increases NADH consumption and NAD^+^ levels, thereby activating SIRT1 and promoting mitochondrial biogenesis 89. It exerts neuroprotective effects by enhancing mitochondrial function in neurons and glial cells, thereby reducing oxidative stress, maintaining mitochondrial integrity, and suppressing mitochondrial apoptosis pathways, thus improving mitochondrial redox homeostasis 90.

As illustrated in Figure 1, the mechanisms of hyperbaric oxygen therapy (HBOT) in neurological disorders can be conceptualized as a coordinated modulation of key pathological processes that converge on cognitive dysfunction. At the neurovascular level, HBOT increases tissue oxygen availability and supports microvascular function and blood-brain barrier homeostasis, potentially through the regulation of angiogenic and trophic mediators (e.g., VEGF) and improved microcirculatory dynamics, thereby enhancing cerebral perfusion and vascular integrity. At the inflammatory and glial-response level, HBOT is associated with attenuated neuroinflammation, including reduced microglial activation and reactive astrocytosis, accompanied by downregulation of pro-inflammatory cytokines such as TNF-α, IL-1β, and IL-6, which may collectively limit secondary neuronal injury. HBOT may exert neuroprotective effects by reducing oxidative stress and mitigating mitochondrial dysfunction. Enhanced antioxidant defenses can constrain ROS accumulation and help preserve neuronal viability. Concurrently, modulation of apoptosis-related pathways may contribute to maintaining neuronal structure and function. Notably, the relative contribution of these mechanisms may vary by disease context, disease stage, and treatment protocol parameters.

To systematically elucidate the mechanisms underlying HBOT in neurological disorders, this paper integrates existing evidence to construct a schematic diagram of its mechanisms (**Figure 1**). This diagram summarizes the common core pathological pathways leading to cognitive impairment in various diseases such as ischemic stroke, Alzheimer's disease, Parkinson's disease, and traumatic brain injury. These primarily include disruption of the blood-brain barrier, neuroinflammation, neuronal loss and degeneration, and oxidative stress. As illustrated, hyperbaric oxygen therapy counteracts these pathological processes through multi-targeted actions: it promotes angiogenesis and axonal repair while exerting anti-inflammatory, anti-apoptotic, and antioxidant effects, thereby achieving neuroprotection at multiple levels. Ultimately, these synergistic actions contribute to comprehensive improvements in attention, memory, and executive function, alleviating symptoms of cognitive impairment.

### Application of Hyperbaric Oxygen Therapy in Disease Models

Because cognitive impairment arises from biologically distinct etiologies, HBOT-related evidence should be interpreted according to disease-specific mechanism-treatment alignment rather than as a uniform effect across all cognitive disorders. In vascular and traumatic cognitive impairment, such as stroke and traumatic brain injury, tissue hypoxia, cerebral hypoperfusion, edema, blood-brain barrier disruption, secondary inflammation, and impaired neuroplasticity are central pathological processes and are relatively well aligned with the physiological actions of HBOT. In contrast, in neurodegenerative cognitive impairment, such as Alzheimer's disease and Parkinson's disease, protein aggregation, progressive neuronal loss, autophagy-lysosomal dysfunction, mitochondrial dysfunction, and gliosis are dominant pathological drivers and are less directly targeted by HBOT. Therefore, the following evidence is discussed according to this etiological and mechanistic distinction.

Hyperbaric oxygen therapy exerts neuroprotective and cognitive-enhancing effects through a multi-target network mechanism. Studies indicate that even in healthy individuals, the hyperoxic environment created by hyperbaric oxygen therapy significantly improves cognitive performance, information processing speed, and decision-making ability during single-task or multi-task execution 91. The following systematically reviews research progress and clinical evidence regarding hyperbaric oxygen therapy in cognitive impairments caused by several major neurological disorders (**Table 2**).

#### Alzheimer's disease

Oxidative stress exacerbates Aβ production and Tau phosphorylation, whilst the accumulation of Aβ and Tau further aggravates redox imbalance, creating a vicious cycle that propels the progression of Alzheimer's disease pathology 92. Existing preclinical studies suggest that HBOT may influence several secondary or modulatory AD-related pathological pathways indirectly, including cerebral oxygenation, microvascular function, oxidative stress, neuroinflammation, and autophagy-related processes. However, these findings should not be interpreted as definitive evidence that HBOT directly reverses the core neurodegenerative pathology 93. Long-term HBOT intervention was associated with reduced hyperphosphorylated Tau aggregation, attenuated microglial and astrocytic activation, and lower levels of TNF-α, IL-6, and IL-1β, suggesting potential modulation of AD-related neuroinflammatory and protein-aggregation pathways 94. A meta-analysis of randomized controlled trials indicated that long-term HBOT was associated with improvements in oxidative stress and inflammatory markers in patients with Alzheimer's disease:malondialdehyde (MDA) [SMD = -2.83, 95% CI (-5.27, -0.38), p = 0.02], superoxide dismutase (SOD) [SMD = 2.12, 95% CI (1.10, 3.15), p < 0.0001], interleukin-1β (IL-1β) [SMD = -1.00, 95% CI (-1.48, -0.53), p < 0.0001] and transforming growth factor-β1 (TGF-β1) [MD = 4.87, 95% CI (3.98, 5.76), p < 0.00001]; and was associated with improved cognitive scores ( ADAS-Cog [MD = -4.53, 95% CI (-5.05, -4.00), p < 0.00001]). Furthermore, this therapeutic effect persisted after the intervention ended 95. Although the results were statistically significant, the magnitude of improvement was only close to or slightly above the minimum clinically important difference (MCID); further validation is needed in conjunction with patients' subjective experiences and larger-scale studies.

#### Stroke

In an acute rat model of middle cerebral artery occlusion (MCAO), hyperbaric oxygen therapy promotes the proliferation of endogenous neural progenitor cells, directs the migration of bone marrow-derived stem cells to the injured brain region, and prevents the loss of immature neurons and dendritic degeneration, thereby facilitating neural circuit reconstruction 96. Similarly, Bao et al. found that hyperbaric oxygen therapy reduces cerebral infarct volume in MCAO mice and alleviates neurological deficits 97.

In clinical studies, focal ischemic hypoxic necrosis is considered the primary mechanism of brain tissue damage in acute ischemic stroke 98. Hyperbaric oxygen therapy improves oxygen supply to damaged brain regions, enhances hypoxic metabolism, and was associated with improvement in neurological deficit scores in elderly patients with acute cerebral infarction (NIHSS score: Cohen's d = 0.610, 95% CI = 1.211-4.658;CSS score : Cohen's d = 1.392, 95% CI = 4.565-7.730;NSE: Cohen's d = 1.976, 95% CI = 4.298-6.203), reduced inflammatory responses (CRP: Cohen's d = 1.353, 95% CI = 9.005-15.376;IL-6: Cohen's d = 3.147, 95% CI = 9.449-11.870), and showed improvement in activities of daily living (P < 0.05, Cohen's d = -0.598, 95% CI = -12.791--2.914).The overall clinical response rate was significantly improved (P < 0.05, OR = 0.335, 95% CI = 0.120-0.932), suggesting potential neurological improvement 99 . However, selection bias may exist due to sample size limitations, and the generalizability of the results requires further evaluation. A retrospective analysis by Hadanny et al. of patients with advanced chronic stroke showed statistically significant improvements in multiple cognitive domains (including memory, executive function, and information processing speed) following hyperbaric oxygen therapy (p < 0.05), with 86% of patients achieving predefined clinically significant improvement (CSI). The study defined CSI as an absolute increase of at least 7.5 points (i.e., 0.5 standard deviations) in the normalized score in at least one cognitive domain 100. This study is subject to information bias and requires validation through future prospective, randomized, blinded controlled trials.

Notably, even when hyperbaric oxygen therapy was administered 3 to 6 months after the onset of ischemic stroke, patients with ischemic stroke demonstrated statistically significant improvements in neurological function (NIHSS score improved from 7.27±2.71 to 5.46±2.47,MMSE score increased from 24.8 ± 2.98 to 26.73 ± 1.9).At the same time, hyperbaric oxygen therapy was associated with improvement in several quality-of-life dimensions as assessed by the SF-36 Quality of Life Questionnaire; except for the physical pain and vitality dimensions, all other dimensions (physical limitations, emotional limitations, energy, social functioning, physical functioning, and general health) showed significant improvement compared to baseline (P < 0.05), which may be related to enhanced brain tissue oxygenation 101 .However, the study has limitations such as a small sample size and a short duration of hyperbaric oxygen therapy intervention (only 24 sessions) , and the reliability of the results should be interpreted with caution.

#### Traumatic brain injury

Hyperbaric oxygen therapy has shown potential neuroprotective and reparative effects in both the acute and chronic phases of traumatic brain injury. By reducing cerebral edema and promoting axonal outgrowth and synaptic remodeling, it may contribute to neurological functional recovery of patients with traumatic brain injury.

During the acute phase (24-48 hours post-injury), hyperbaric oxygen therapy can modulate mitochondrial permeability, upregulate serum glutathione levels—a marker of oxidative stress—and maintain these levels for at least eight weeks, thereby alleviating the secondary damage cascade associated with traumatic brain injury 102 .A study using an explosion-induced traumatic brain injury rabbit model found that hyperbaric oxygen therapy reduced the area of the penumbra surrounding contusions, alleviated cerebral edema, maintained blood-brain barrier integrity, and inhibited neuronal edema, inflammatory cell infiltration, and apoptosis 103 . During the chronic phase of traumatic brain injury, hyperbaric oxygen therapy has been shown to improve spatial learning and memory in rats with traumatic brain injury 104. Klaus Kraitsy et al. found that a three-week course of hyperbaric oxygen therapy promotes myelin regeneration in rats with traumatic brain injury (05), and clinical evaluations indicate that increased white matter fiber integrity is associated with improved cognitive function 106. Imaging studies showed that HBOT was associated with angiogenesis-related and microstructural changes in patients with chronic traumatic brain injury, including increased blood volume and microstructural integrity in key cognitive brain regions such as the frontal lobe white matter, anterior cingulate cortex, thalamus, and hippocampus, as well as reduced abnormal slow waves on electroencephalograms 107.

A meta-analysis based on the NeuroTrax cognitive assessment system showed that hyperbaric oxygen therapy was associated with improvements in several cognitive domains in patients with traumatic brain injury, including memory (MD = 10.13, P < 0.0001), attention (MD = 7.99, P < 0.00001), executive function (MD = 7.16, P = 0.002), information processing speed (MD = 7.48, P = 0.01), and motor skills (MD = 5.19, P < 0.00001) 108. Furthermore, even decades after the initial trauma, hyperbaric oxygen therapy may support neuroplasticity and cognitive recovery in selected patients in post-concussion syndrome 109. At the molecular level, hyperbaric oxygen therapy can upregulate levels of neurotrophic factors such as brain-derived neurotrophic factor (BDNF), nerve growth factor (NGF), and VEGF, which may be one of the key mechanisms underlying its promotion of traumatic brain injury recovery 110.

#### Other neurological disorders

Compared with stroke and traumatic brain injury, evidence for HBOT in other neurological disorders remains more heterogeneous and is often derived from animal studies, small clinical studies, or observational analyses. Therefore, these findings should be interpreted as preliminary and hypothesis-generating.

In a sepsis-associated encephalopathy model, hyperbaric oxygen therapy reduced the expression of HIF-1α and IL-6 in peripheral blood and hippocampal tissue, decreased microglial activation, improved cognitive function, and increased survival rates 111. For acute spinal cord injury, hyperbaric oxygen therapy was reported to alleviate tissue hypoxia, reduce inflammatory responses, improve neuronal survival rates, and support respiratory function recovery 112. Similarly, in a model of radiation-induced brain injury, hyperbaric oxygen therapy was reported to alleviate radiation-induced cognitive deficits by increasing the proliferation of neurogenesis in the hippocampal dentate gyrus and reducing lipid peroxidation 113.

Delayed cerebral ischemia, a common complication of aneurysmal subarachnoid hemorrhage, is often accompanied by cognitive decline and severely impedes neural repair. Hyperbaric oxygen therapy can attenuate the elevation in vasospasm index and arterial wall thickening induced by subarachnoid hemorrhage 114. Patients who received hyperbaric oxygen therapy early showed a significantly lower incidence of delayed cerebral ischemia (P = 0.026), and the long-term reconstruction of the attention network (particularly, which is involved in executive control functions) was also reported (P < 0.001) 115. On the other hand, HBOT was associated with improved coupling of cerebral circulation and metabolism, improvement in Glasgow Coma Scale (GCS) and NIHSS scores (P < 0.05), and improved consciousness and prognosis in patients with intracerebral hemorrhage 116. Furthermore, nearly one-third of COVID-19 patients experience long-term neurological symptoms, including “brain fog,” cognitive impairment, anxiety, depression, and sleep disorders. High antioxidant activity has been shown to help enhance protection against SARS-CoV-2 infection 117. Addressing mitochondrial dysfunction is a key component of mitigating the long-term neurological effects of COVID-19. Following hyperbaric oxygen therapy, improvements were reported in memory, executive function, attention, fatigue, and pain, and no major safety signals were reported in that study 118.

## Discussion on Treatment Heterogeneity and Beneficiary Populations

Although hyperbaric oxygen therapy has significantly alleviated the cognitive impairments caused by the aforementioned conditions, its efficacy is not universally applicable due to fundamental differences in the degree to which the core pathophysiological mechanisms underlying different etiologies align with the mechanism of action of hyperbaric oxygen therapy. Based on current evidence, the efficacy of this treatment follows a clear pattern: vascular and traumatic cognitive impairments are the areas where the benefits of hyperbaric oxygen therapy are most evident and where the evidence is relatively robust (e.g., stroke, traumatic brain injury) 119,120. The common core pathological mechanisms in these conditions involve focal or diffuse cerebral hypoxia, insufficient perfusion, edema, and secondary inflammation—pathological processes that hyperbaric oxygen therapy can directly target through multiple mechanisms. Consequently, studies involving such patients often demonstrate consistent improvements in cognitive function scales, activities of daily living, and cerebral perfusion or metabolic imaging indices 121,122.

In stark contrast, the therapeutic effects for neurodegenerative cognitive disorders (such as Alzheimer's disease and Parkinson's disease) are relatively modest, with significant individual variability, and symptoms are often only partially alleviated 123. Early intervention yields relatively clear benefits, whereas outcomes in the middle and late stages are poorer. Direct neurotoxicity resulting from the accumulation of misfolded proteins is a hallmark of most neurodegenerative diseases, typically involving pathological processes such as mitochondrial dysfunction, autophagy-lysosomal dysfunction, neuronal death, and gliosis; however, these primary pathological targets are not the direct targets of hyperbaric oxygen therapy 124-126 .Hyperbaric oxygen therapy may exert potential neuroprotective or symptom-relieving effects indirectly by improving the whole-brain or local microenvironment (e.g., reducing neuroinflammation, alleviating oxidative stress, and enhancing cerebral blood flow) 127 . Therefore, the greater the overlap between the core pathological mechanisms of a disease and the known core physiological mechanisms of hyperbaric oxygen therapy, the clearer and more significant the clinical benefits tend to be.

In summary, we can preliminarily outline the characteristics of the “optimal patient population” likely to derive the greatest clinical benefit from hyperbaric oxygen therapy; clinical treatment should involve a differentiated assessment based on the specific etiology. 1. Within the reversible therapeutic window (primarily applicable to vascular/traumatic etiologies): Patients in the acute or subacute phase (typically within 6-12 months after the initial event) whose neurological deficits are still dynamically changing and whose brain plasticity remains strong. During this phase, timely correction of hypoxia and secondary damage through hyperbaric oxygen therapy is most likely to rescue neurons within the ischemic penumbra (rather than protein deposits such as Aβ plaques) and promote neural network remodeling 128. 2. Presence of clear biological treatment targets (primarily applicable to vascular/traumatic etiologies): Neuroimaging evidence reveals focal areas of perfusion deficiency, ischemia, or reduced metabolism corresponding to clinical symptoms, commonly observed in patients with acute ischemic stroke or focal cerebral contusion. Such evidence provides objective intervention targets for hyperbaric oxygen therapy and enables more targeted efficacy assessment 129. 3. Good functional reserve (applicable to all etiologies, though with varying weighting): Patients who are relatively young and have well-preserved baseline cognitive function and activities of daily living (ADL) possess greater brain repair potential and are more likely to translate physiological improvements into meaningful clinical functional gains. This characteristic is relatively limited in neurodegenerative etiologies due to the progressive nature of the disease.4. Vascular components in mixed etiologies (primarily applicable to specific subgroups within neurodegenerative diseases): In patients diagnosed with neurodegenerative diseases, the presence of significant evidence of cerebrovascular disease (e.g., hyperintensities in white matter, lacunar infarcts) suggests that the vascular component of their cognitive impairment may respond to hyperbaric oxygen therapy, leading to partial symptom improvement 130,131. It is important to note that the mechanism of benefit in these patients does not involve direct action on pathological targets, but rather through the improvement of coexisting cerebral hypoperfusion and hypoxic microenvironments. Therefore, even if effective, the therapeutic effect is limited to the vascular component and does not reverse the neurodegenerative pathology itself.

By identifying and prioritizing treatment for the subgroups most likely to benefit, we can shift from broad-spectrum validation of disease efficacy to precision medicine-based patient stratification strategies, ultimately propelling hyperbaric oxygen therapy into a new era of individualization and precision in the field of neurological disorders. For patients with poor or incomplete responses, a key direction for future research is to evaluate whether combining hyperbaric oxygen therapy with other treatments can overcome efficacy bottlenecks and expand the population that benefits.

### Limitations and future prospects of hyperbaric oxygen therapy

Despite substantial preclinical and preliminary clinical evidence demonstrating hyperbaric oxygen therapy's promising potential for improving cognitive impairment, establishing it as a standard treatment protocol remains challenging. These obstacles clearly delineate future research directions.

Firstly, the depth and systematic nature of mechanism research require urgent enhancement. Current understanding of hyperbaric oxygen therapy's mechanisms remains fragmented, with the intrinsic connections and temporal patterns between its induced effects—including angiogenesis, neurogenesis, anti-inflammation, and antioxidant actions—yet to be elucidated. Future work must employ high-throughput technologies such as spatial transcriptomics, proteomics, and metabolomics, combined with pathway-specific regulatory tools, to systematically decipher the precise molecular targets and signaling networks of hyperbaric oxygen therapy across diverse disease models and disease stages. This will enable the construction of a comprehensive mechanistic map.

Secondly, the implementation plan—transitioning from standardized and individualized approaches to combined therapies—is pivotal for clinical translation. Standardized parameters form the foundation for ensuring research reproducibility and comparability. Currently, significant variations exist in hyperbaric oxygen parameters (treatment pressure, single-session duration, frequency, and total course length) across studies. This heterogeneity hinders direct comparison of results between studies and prevents high-quality meta-analyses, severely undermining the efficiency of accumulating evidence. For clinicians, parameter standardization ensures treatments remain within safe and effective parameters, providing clear grounds for developing clinical practice guidelines, training manuals, and insurance reimbursement standards. This represents not only an ethical imperative for patient safety and standardized care but also the practical foundation for reliable, accessible implementation across broader healthcare settings. A major challenge in the clinical translation of hyperbaric oxygen therapy is the substantial heterogeneity in treatment parameters, including chamber pressure, session duration, treatment frequency, and total course length. Such variability not only hampers meaningful comparisons across studies but also obscures potential dose-response relationships, thereby limiting the development of standardized therapeutic protocols. Importantly, optimization of hyperbaric oxygen therapy should focus on identifying appropriate therapeutic windows rather than simply maximizing oxygen exposure. Although no unified clinical guidelines currently exist for the application of hyperbaric oxygen therapy in cognitive impairment, an increasing number of studies have begun to explore associations between treatment pressure, session number, and therapeutic efficacy. Emerging evidence suggests that protocol optimization should be tailored according to disease type, disease stage, and therapeutic objectives, such as acute neuroprotection versus chronic neuroplastic remodeling. Future investigations specifically designed to elucidate dose-response relationships—through systematic modulation of treatment pressure, session frequency, and total treatment duration—are critically needed to establish evidence-based, disease-specific, and stage-adapted hyperbaric oxygen therapy protocols.

It is important to note that standardization does not pursue a rigid, one-size-fits-all approach but aims to establish a systematic research framework. Therefore, based on existing evidence, we propose personalized guiding principles to optimize current protocols for future research reference: 1. Gradually move away from traditional methods that solely describe “pressure-time” and introduce metrics like “atmosphere-minutes” or similar indicators to scientifically quantify cumulative oxygen exposure dose. Given that different disease pathologies (vascular, degenerative, traumatic) may determine their dose sensitivity and safety thresholds, single-disease dose-escalation Phase I/II clinical trials could be designed to map preliminary disease-specific response curves. 2. Define optimal treatment windows for different diseases. Future randomized controlled trials incorporating diverse parameter combinations are urgently needed to identify efficacy thresholds and plateau phases for key parameters—particularly pressure and total treatment sessions. Pressure likely primarily determines oxygen diffusion depth, while total sessions may correlate with efficacy persistence. Independent and interactive effects of these parameters require exploration. 3. Phased treatment: Drawing from existing clinical practice, a “basic induction phase” + “consolidation maintenance phase” framework may be adopted. For example, initiate 20-40 concentrated treatments to break the vicious cycle of hypoxia and induce physiological effects, followed by intermittent maintenance therapy based on response. 4. Dynamic adjustment: During treatment, functional assessments should be conducted regularly based on disease type, stage, severity, and individual response to adjust subsequent treatment plans accordingly. 5. Combine multimodal assessment tools to identify objective indicators capable of predicting and monitoring individualized treatment responses. Examples include baseline cerebral blood flow perfusion characteristics, specific inflammatory cytokine profiles (e.g., IL-6, TNF-α), neuroimaging markers (e.g., white matter integrity), biomarkers, or genetic background (e.g., ApoE genotype). This will facilitate the identification of high-response populations, enabling precision medicine to optimize healthcare resource allocation and enhance cost-effectiveness.

Exploring combined strategies of hyperbaric oxygen therapy with existing treatments represents a key direction for overcoming efficacy bottlenecks and achieving synergistic enhancement. Current clinical management of cognitive impairment already advocates for multimodal interventions. Existing research indicates that the combination of atorvastatin and hyperbaric oxygen therapy can regulate cerebral hemodynamics, reduce brain tissue damage, and significantly enhance cognitive and limb recovery capabilities, demonstrating immense value in early traumatic brain injury rehabilitation 132. The combined use of acupuncture and hyperbaric oxygen therapy not only significantly reduced levels of brain injury markers (homocysteine and vesinic-like protein-1) and oxidative stress indicators (malondialdehyde and 8-oxo-2'-deoxyguanosine), but also markedly altered the amplitude and latency of the P300, a cognitive function marker, while elevating levels of neuroprotective factors (insulin-like growth factor, BDNF). By enhancing effective blood diffusion rate, improving brain metabolism, and inducing neuronal remodeling, it significantly ameliorates post-stroke cognitive impairment, yielding superior therapeutic outcomes compared to hyperbaric oxygen therapy alone 133. The combination of hyperbaric oxygen therapy and music therapy significantly improves brain neural deficits, slows cerebral arterial blood flow, promotes brain function recovery in patients with postoperative aneurysmal subarachnoid hemorrhage, alleviates anxiety and depression, and enhances patients' activities of daily living scores 134. Following long-term hyperbaric oxygen therapy combined with intravenous corticosteroids, speech therapy, and physical rehabilitation, it significantly improves cognitive impairments caused by delayed neurological sequelae 135. The aforementioned combined treatment strategies hold immense potential for enhancing cognitive function, promoting neuroprotection, and facilitating patient recovery. Future research should elucidate the mechanisms underlying these synergistic effects and validate them clinically, thereby establishing a patient-centered, personalized integrated treatment system.

Finally, the level of evidence requires higher-grade clinical studies to be elevated. Although current evidence suggests the potential efficacy of hyperbaric oxygen therapy, it must be objectively acknowledged that the existing clinical evidence base has certain limitations. First, most studies have small sample sizes (n < 50), which may lead to insufficient statistical power, making it difficult to detect subtle yet clinically meaningful differences and increasing the risk of results being influenced by chance factors or individual variability. Second, study designs predominantly rely on observational approaches (e.g., case series) or non-randomized controlled trials. Inherent selection bias, measurement bias, and confounding factors in such designs are difficult to fully control, potentially compromising the precision of efficacy estimates and limiting the strength of causal inferences. Consequently, current evidence is more appropriately viewed as exploratory, generating hypotheses and indicating directions, rather than definitive conclusions.

Future RCTs should incorporate adequate sample sizes powered to detect clinically meaningful cognitive changes, preferably based on standardized neuropsychological outcome measures. The inclusion of sham-controlled and double-blinded designs is particularly critical in hyperbaric oxygen therapy research, given the strong expectancy and placebo effects associated with chamber-based interventions. In addition, stratification by disease subtype, disease stage, and baseline cerebral perfusion or neuroimaging characteristics may help reduce heterogeneity and identify responder subgroups.

To build a more robust and generalizable evidence base, future research should prioritize large-scale, methodologically rigorous RCTs. Specific requirements include: 1) Study Design: Employ multicenter, randomized, double-blind, placebo-controlled (e.g., using simulated pressurized air) superiority or non-inferiority designs to confirm efficacy; 2) Sample size: Conduct prior sample size calculations based on clinically meaningful minimum differences, fully accounting for attrition during follow-up to ensure sufficient statistical power (e.g., ≥ 80%); 3) Endpoints: Establish patient-centered, well-validated cognitive assessment tools (e.g., ADAS-Cog, MoCA) as primary endpoints, while incorporating neuroimaging changes (e.g., fMRI, DTI), blood biomarkers, and quality-of-life scores as secondary endpoints to comprehensively elucidate treatment mechanisms and clinical benefits; 4) Protocol Standardization and Long-Term Follow-Up: Develop and report detailed standardized treatment protocols, with sufficient follow-up periods (e.g., 6-12 months or longer) to assess sustained efficacy and long-term safety.

Through deepening mechanism research and establishing standardized, individualized, and combined treatment protocols, robust evidence-based medical support will be obtained. Hyperbaric oxygen therapy holds promise to transcend its traditional adjunctive role. It may evolve into a key component within the comprehensive prevention and management system for cognitive impairment in neurological disorders - one characterized by clear mechanisms, precise protocols, and substantial evidence - offering renewed hope for improving patients' long-term prognosis.

## Conclusion

In summary, cognitive impairment—a common core symptom across multiple neurological disorders—reveals a pathological pathway of "hypoxia-neurovascular unit imbalance-neural network degeneration" that provides critical theoretical entry points and intervention targets for hyperbaric oxygen therapy. Evidence reviewed herein indicates that hyperbaric oxygen therapy exerts its efficacy through multi-targeted, synergistic mechanisms, demonstrating promising prospects for improving cognitive function in neurological disorders. While limitations persist in clinical application, hyperbaric oxygen therapy holds future potential as a vital component within comprehensive cognitive impairment prevention and management systems, offering novel hope and therapeutic strategies for affected patients.

## Figures and Tables

**Figure 1:**
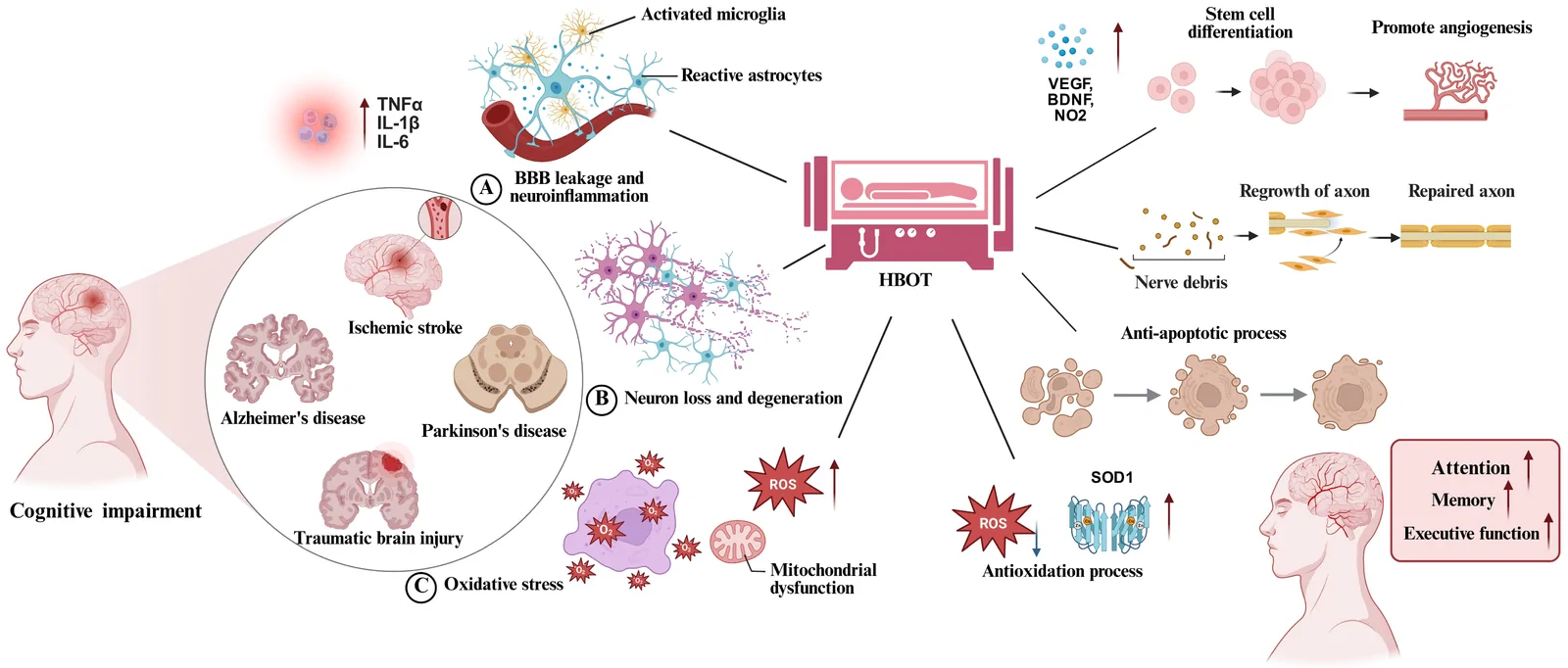
Mechanism diagram of how hyperbaric oxygen improves cognitive function.

**Table 1:** Mechanisms by which hyperbaric oxygen therapy improves cognitive function

HBOT-related physiological trigger / level of action	Immediate biological effect	Key molecules/pathways	Primary biological effects and cognitive-related outcomes
Increased oxygen partial pressure and enhanced oxygen diffusion affecting the blood-brain barrier and cerebral blood flow	Maintains blood-brain barrier integrity; dilates blood vessels to increase cerebral blood flow; reduces cerebral edema and necrosis	VEGF (62,63); bFGF; HIF-1α; NO; NOS	Maintains blood-brain barrier integrity (61). Dilates blood vessels to increase cerebral blood flow. Reduces cerebral edema and necrosis (64). Promotes angiogenesis and alleviates local hypoxia (65) (66).
Oxygen- and pressure-sensitive gene regulation	Increases stem cell mobilization and creates a microenvironment favorable for neuroregeneration	mTOR (68); p62; Beclin-1; LC3; Bcl-2; BAX	Regulates oxygen/pressure-sensitive genes to increase stem cell mobilization (67). Promotes autophagosome formation and lysosomal fusion; clears toxic protein aggregates to activate the autophagy process (69). Restores the Bcl-2/BAX balance to reduce neuronal apoptosis (70).
Enhanced neurotrophic signaling and synaptic remodeling	Promotes hippocampal long-term potentiation and synaptic plasticity	CREB (71); BDNF (72); TrkB; MAPK/ERK; PI3K/Akt	Promotes hippocampal LTP (73). Promotes the transcription of plasticity genes (74). Enhances synaptic transmission and cognitive function (75).
Improved oxygenation and repair of white matter microenvironment	Enhances cerebral blood flow and promotes clearance of myelin debris	Macrophages; Schwann cells; Neurotrophic factors	Enhances cerebral blood flow and increases macrophage activity to clear myelin debris. Activates Schwann cells to release neurotrophic factors, accelerating axon and myelin regeneration (77,78). Improves information processing speed and episodic memory (79).
Suppression of glial inflammatory activation	Repairs the blood-brain barrier; inhibits abnormal proliferation of astrocytes and microglia; reduces inflammatory cytokine production	IκBα; NF-κB; IL-1β; IL-6; TNF-α; Bcl-2	Repairs the blood-brain barrier and inhibits abnormal proliferation of astrocytes and microglia; Upregulates Bcl-2 to inhibit apoptosis. Stabilizes IκBα, inhibits the NF-κB pathway, and reduces the production of pro-inflammatory factors (82-84).
Intermittent hyperoxia-induced redox modulation	Enhances antioxidant enzyme activity and increases total antioxidant capacity	SOD1/2; glutathione peroxidase (GPx); ROS	Enhances antioxidant enzyme activity and increases total antioxidant capacity (88). Reduces ROS levels to prevent lipid peroxidation, protein damage, and DNA damage.
Improved mitochondrial oxygen utilization and mitochondrial homeostasis	Increases NADH consumption and elevates NAD⁺ levels	NADH/NAD⁺; SIRT1; Mitochondria	Increases NADH consumption, elevates NAD⁺ levels, and activates SIRT1 to promote mitochondrial biogenesis (89). Alleviates oxidative stress, maintains mitochondrial integrity, and inhibits mitochondrial apoptosis pathways (90).

**Table 2:** Application and evidence of hyperbaric oxygen therapy in cognitive impairment caused by different neurological diseases

Disease Type	Study Design/Population	Evidence of Validity	Level of Evidence	Sample Size
Alzheimer's Disease	5XFAD transgenic AD Mice (93)	Was associated with improved cerebral oxygenation and cerebral blood flow; repaired small artery structure; associated with reduced Aβ burden; associated with improved behavioral performance	Level IV evidence Animal studies	*n* = 10/group for key analyses; elderly subgroup *n* = 6
	APPswe/PS1dE9 Double-transgenic mice (94)	Was associated with reduced aggregation of hyperphosphorylated tau protein; attenuated abnormal activation of microglia and astrocytes; lowered levels of TNF-α, IL-6, and IL-1β; associated with improved cognitive performance	Level IV evidence Animal studies	48 mice total
	Alzheimer's disease Patients (95)	Was associated with reduced oxidative stress markers (MDA, SOD) and inflammatory markers (IL-1β, TGF-β1); associated with improved ADAS-Cog scores, with effects reported after intervention cessation	Level I evidence Meta-analysis of randomized controlled clinical trials	11 RCTs; *n* = 847
Stroke	MCAO models in rats and mice	Promotes proliferation of endogenous neural progenitor cells, directs stem cells to migrate to damaged brain regions, and prevents loss of immature neurons and dendritic degeneration (96); Reduces cerebral infarct volume and alleviates neurological deficits (97)	Level IV evidence Animal studies	32 rats
	Elderly patients with acute cerebral infarction (99)	May improve oxygen supply to damaged brain regions and enhance hypoxic metabolism; was associated with improvement in neurological deficit scores (NIHSS score, CSS score, NSE); reduced inflammatory responses (CRP, IL-6); showed improvement in activities of daily living	Level I evidence Randomized controlled trials	122 patients
	Patients with late-stage chronic stroke (100)	Was associated with improvement in selected cognitive domains, including memory, executive function, and information processing speed.	Level II evidence Large-scale retrospective analysis	162 patients
	Patients with ischemic stroke 3 to 6 months post-onset (101)	Was associated with enhanced cerebral oxygenation and improvements in neurological function (NIHSS score, MMSE score) and quality-of-life dimensions	Level I evidence Randomized controlled pilot trial	30 patients
	Patients with acute traumatic brain injury (102)	Was associated with regulation of mitochondrial permeability and increased serum glutathione levels; may alleviate the secondary damage cascade	Level III evidence Small-sample prospective study	40 patients
	Rabbit blast-induced traumatic brain injury model (103)	Was associated with reduced contusion penumbra area, alleviated cerebral edema, and maintained blood-brain barrier integrity; reduced neuronal edema, inflammatory cell infiltration, and apoptosis	Level IV evidence Animal studies	Not clearly reported
	Rat model of chronic traumatic brain injury	Was associated with improved spatial learning and memory (104); promoted myelin regeneration (105)	Level IV evidence Animal studies	Ref. 104: 64 rats; Ref. 105: n ≥ 7-13
Traumatic brain injury	Patients with chronic traumatic brain injury (107)	Was associated with angiogenesis-related and microstructural changes; increased blood volume and microstructural integrity in key cognitive brain regions such as the frontal white matter, anterior cingulate cortex, thalamus, and hippocampus; reduced abnormal slow waves on EEG	Level III evidence Small-sample retrospective analysis	15 patients
	Patients with traumatic brain injury (108)	Was associated with improvements in selected cognitive domains, including memory, attention, executive function, information processing speed, and motor skills	Level I evidence Meta-analysis of clinical randomized controlled trials	Not clearly reported
	Patients with post-concussion syndrome (109)	May support neuroplasticity and cognitive recovery	Level II evidence Retrospective cohort studies	26 adults
Other neurological disorders	Mouse model of sepsis-associated encephalopathy (111)	Was associated with reduced expression of HIF-1α and IL-6 in peripheral blood and hippocampal tissue, decreased microglial activation, improved cognitive performance, and increased survival rate	Level IV evidence Animal studies	Not clearly reported
	Acute spinal cord injury model (112)	Was reported to alleviate tissue hypoxia, reduce inflammation, increase neuronal survival, and support respiratory function recovery	Level IV evidence Animal studies	Not clearly reported
	Rat Model of Radiation-Induced Brain Injury (113)	Was reported to increase proliferation of new neurons in the hippocampal dentate gyrus, reduce lipid peroxidation, and alleviate radiation-induced cognitive deficits	Level IV evidence Animal studies	24 rats total; *n* = 6/group
	Patients with aneurysmal subarachnoid hemorrhage Patients with concomitant delayed cerebral ischemia (115)	Early administration was associated with a reduced incidence of delayed cerebral ischemia and long-term reconstruction of the attention network	Level II evidence Large-scale prospective studies	98 patients; 25 healthy controls
	Patients with intracerebral hemorrhage (116)	Was associated with improved coupling of cerebral circulation and metabolism, improvement in GCS and NIHSS scores, and improved level of consciousness and prognosis	Level I evidence Systematic review	Not clearly reported
	Patients with COVID-19 (118)	Reported improvements in memory, executive function, attention, fatigue, and pain	Level I evidence Systematic review	Not clearly reported
